# PDMS-Encapsulated MXene@Polyester Fabric Strain Sensor for Multifunctional Sensing Applications

**DOI:** 10.3390/nano12050871

**Published:** 2022-03-05

**Authors:** Wengang Lu, Beenish Mustafa, Zhiyuan Wang, Fuzhuo Lian, Geliang Yu

**Affiliations:** 1 National Laboratory of Solid State Microstructures, School of Physics, Nanjing University, Nanjing 210093, China; wengang.lu@foxmail.com (W.L.); dg1822501@smail.nju.edu.cn (B.M.); wzy10575@163.com (Z.W.); 15850786328@163.com (F.L.); 2 Collaborative Innovation Centre of Advanced Microsctructures, Nanjing University, Nanjing 210093, China

**Keywords:** polyester, MXene, strain sensor, human motions, sound detection, PDMS

## Abstract

Flexible strain sensors based on 2D materials have been proven effective for wearable health monitoring devices, human motion detection, and fitness applications. These sensors are flexible, light, and user-friendly, but their sensitivity and detection range need to be enhanced. Among many 2D materials, MXene attracts much interest due to its remarkable properties, such as high electrical conductivity, excellent mechanical properties, flexibility, and good hydrophilicity. However, it is a challenge to fabricate strain sensors with extreme sensitivity and a wide sensing range. In this work, a multifunctional, cost-effective, and highly sensitive PDMS-encapsulated MXene@polyester fabric strain sensor was fabricated. Firstly, complete adsorption of MXene within the fabric formed conductive networks, and then PDMS was used to endow superhydrophobicity and corrosion resistance. The strain sensor demonstrated multifunctional applications and outstanding performance, such as long-term stability (over 500 cycles) and a wide sensing range (8%). The proposed sensor has promising potential for wearable electronic devices such as health monitoring systems and physiological sensing applications.

## 1. Introduction

Stretchable and wearable strain sensors have generated significant interest in various applications, from skin detection to health monitoring systems [1,2,3,4,5,6]. The sensors have been attached to different body parts and monitor movements such as breathing, heartbeat, pulse, and body posture [7,8,9]. Wearable stress and strain sensors are becoming more popular as the development of intelligent electronic gadgets accelerates. However, obtaining high-performance strain sensors with high sensitivity, a wide strain range, and a low detection limit remains a major challenge. As a result, much work needs to be done to enhance manufacturing procedures and materials.

Flexible strain sensors based on fabric have obvious advantages in flexible electronic equipment [10,11,12,13]. These are easy to fabricate; inexpensive; and resistant to bending, stretching, torsion, and other complex deformations. Many strain sensors have been fabricated using conductive nanomaterials such as graphene [14,15,16], MXene [11,17,18,19], and silver nanowires [20]. In particular, MXene has been intensively explored for strain sensing applications [21]. MXene is a class of 2D materials with high electrical conductivity, excellent mechanical properties, and specific capacitance. The chemical formula of MXene is M_n+1_X_n_T_x_, where M stands for a transition metal, X for carbon or nitrogen, and T_x_ for terminating functional groups (e.g., OH, -O, -F) [22,23,24]. Among more than 30 types of MXene, Ti_3_C_2_T_x_ MXene is widely used in energy storage [25], sensing devices [26], and electromagnetic interference shielding [27,28]. For example, Yu et al. designed a polyester fabric strain sensor by using 80% polyester and 20% TPU elastic fabric (a weft insertion and warp knitting structure) which shows a broader response range (over 50%), high sensitivity, and good cycle stability [29]. Noticeably, the sensor lacks a protective layer, which could oxidize MXene and make it unstable, reducing its performance. Thus, although textiles are suitable carriers for flexible sensors because of their flexibility, breathability, and skin comfort, their high moisture absorption can limit their wide range of applications in MXene-based sensors. Previous studies have successfully demonstrated that PDMS has the ability to protect MXene from oxidation and reduce the influence of external humidity on sensor performance [18,30,31]. Therefore, using PDMS as a protective layer could be a promising strategy for strengthening MXene-based sensors. Furthermore, data on the efficiency of the suggested sensor in various stretching directions are limited, demanding further investigation.

In this paper, a highly sensitive PDMS-encapsulated MXene@polyester fabric (PMPF) strain sensor was prepared. The sensor is composed of three parts: an elastic polyester fabric substrate, a functional MXene network, and a protective layer of polydimethylsiloxane (PDMS). A Ti_3_C_2_T_x_ MXene nanosheet was synthesized by the MILD method and uniformly deposited on comfortable polyester fabric by electrostatic interaction. PDMS was used to protect MXene from oxidation and reduce the influence of external humidity on sensor performance. Furthermore, as the strain on the PMFP sensor varies, the fabric network structure effectively adjusts the contact area between conductive MXene channels, increasing the strain sensor’s sensing performance. In particular, MXene fabrics contain both wale and course fibers that form conductive networks during stretching and exhibit different tensile properties. As a result, the PMPF strain sensor displayed the comprehensive advantages of an 8% strain range, high sensitivity, and good stability.

## 2. Materials and Methods

### 2.1. Synthesis of Ti_3_C_2_T_x_ MXene

Ti_3_C_2_T_x_ MXene was synthesized using the MILD method [22]. First, 1.6 g of lithium fluoride (LiF) (99.99%, purchased from RhawnCo., Ltd., Shanghai, China) was dissolved into 20 mL of a hydrochloric acid (HCl) solution (12 M) in a Teflon beaker. Then, 1 g of Ti_3_AlC_2_ (purchased from Rhawn Co., Ltd., Shanghai, China) was slowly added to the mixture solution. The mixture was kept stirring at 40 °C for 24 h and washed twice using 2 M HCl. The suspension was then centrifuged and washed with deionized water until the pH of the solution reached around 7. Next, the suspension was sonicated for 2 h (ultrasonic probe, 200 W). Finally, the mixture was centrifuged at 3500 rpm for 1 h, and the supernatant that consisted of Ti_3_C_2_T_x_ MXene dispersion was collected.

### 2.2. Fabrication of PMPF Strain Sensor

First, the polyester fabric (97% polyester, 3% spandex, with a thickness of 0.33 mm) was cut into a rectangular shape (5 cm × 1 cm) along the course direction and wale direction, and the cut fabric was washed with DI water to remove the impurities and then dried thoroughly. Next, the cleaned polyester fabric was dipped into a solution of MXene for three minutes and then dried in an oven at 70 °C for 30 min. Subsequently, we repeated the experimental procedure one more time to ensure the complete absorption of MXene within the fabric. After MXene@polyester preparation, the copper foil was connected at both ends with the help of conductive silver paste (the distance between the two electrodes was 30 mm) and immersed in PDMS solution (a 10:1 mixture of PDMS monomer and curing agent) for three minutes, and then it was taken out and dried in an oven at 70 °C for one hour.

### 2.3. Characterization

The morphologies of all samples were characterized by a scanning electron microscope (S-3400N, Hitachi, Tokyo, Japan) and a transmission electron microscope (Tecnai F20, FRI, Ames, IA, USA). The diffraction peaks of Ti_3_AlC_2_, Ti_3_C_2_T_x_, polyester fabric, and MXene@polyester fabric were obtained by X-ray diffraction (X’TRA) equipment with Cu Kα1 radiation (λ = 0.154 nm) with a step size of 0.02° from 3 to 80°. Thermogravimetric analysis (TGA) (Pyris 1 DSC, PerkinElmer, Waltham, MA, USA) experiments were performed under an argon atmosphere between 100 and 600 °C at a heating rate of 20 °C/min. The chemistry attributes of samples were analyzed by X-ray photoelectron spectroscopy (PHI5000VersaProbe, ULVAC-PHI, Chigasaki, Japan). The electrical signals of the PMPF strain sensor were recorded with a source meter (2614B, Keithley, Solon, OH, USA).

### 2.4. Sensing Performance Measurement

As shown in Figure S1, we fixed the sensor at both ends of the stepping motor motion control platform and connected the copper electrodes at both ends of the sensor with the positive and negative poles of a source meter (2614B, Keithley, Solon, OH, USA). When testing the sensitivity, we set a constant voltage of 0.1 V at both ends of the sensor and controlled one end of the motion platform to move forward at the speed of 0.1 mm/s, giving the sensor tensile strain. During this process, the current change of the sensor was recorded continuously by a source meter, and then the current signal was converted into a resistance signal by Ohm’s law. The slope of the relative resistance change–strain curve is the sensitivity of the sensor within the strain range. Similarly, when testing the frequency response, keeping the voltage unchanged at 0.1 V, the movement distance of the motion platform was set as 5% of the strain of the sensor; the movement speed was set according to the frequencies of 0.1, 0.15, 0.2, 0.25, and 0.3 Hz; and a certain number of cycles were performed at each movement speed. The current changes in the process were recorded and converted into resistance signals and corresponding curves were drawn. When testing the cyclic stability, the voltage at both ends of the sensor was set as 0.1 V, one end of the motion platform moved at a speed of 0.75 mm/s, and the distance was 5% of the strain of the sensor; after repeatedly moving 500 times, the current change was recorded and then converted into the resistance signal, and the cyclic test curve was drawn.

## 3. Results and Discussion

**Figure 1:**
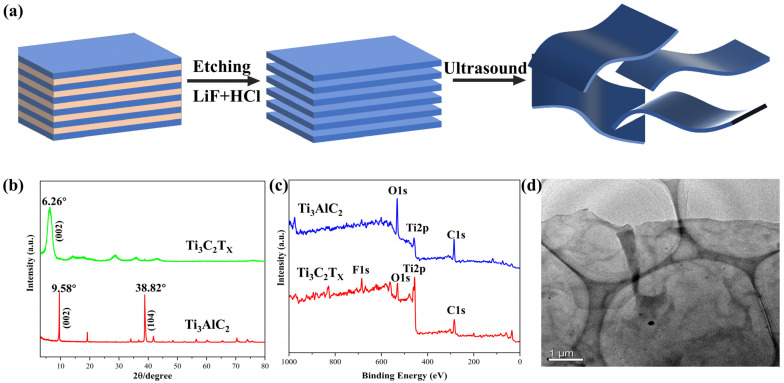
MXene synthesis configuration. (**a**) MXene preparation schematic diagrams; (**b**) XRD of Ti_3_AlC_2_ and Ti_3_C_2_T_x_ nanosheets; (**c**) the XPS patterns of Ti_3_AlC_2_ and Ti_3_C_2_T_x_; (**d**) transmission electron microscopy (TEM) image of Ti_3_C_2_T_x_ nanosheets.

We used a minimally intense layer delamination (MILD) approach to make Ti_3_C_2_T_x_ MXene, as shown in Figure 1a. A series of systematic characterization techniques were carried out to verify the successful preparation of Ti_3_C_2_T_x_ MXene nanosheets and PMPF sensors. Figure 1b shows the XRD pattern of Ti_3_AlC_2_ and exfoliated MXene nanosheets. The characteristic peaks of Ti_3_AlC_2_ at 9.58 and 38.82° are located at (002) and (104) crystal planes, respectively. The strong peak (104) almost completely disappeared after the etching and delamination process, which indicates that the aluminum (Al) atomic layer of Ti_3_AlC_2_ was etched successfully. The angle of the (002) peak of MXene is 6.26° lower than that of Ti_3_AlC_2_ (9.58°), indicating that the distance between layers is extended after the etching and delamination process. The sharpness of the (002) peak is well maintained, showing the prepared MXene has a higher crystallinity and an ordered structure. The surface chemical environments of Ti_3_AlC_2_ and synthesized Ti_3_C_2_T_x_ MXene nanosheets were characterized by X-ray photoelectron spectroscopy (XPS). The elemental composition and surface chemical environment of Ti_3_AlC_2_ and Ti_3_C_2_T_x_ were studied by XPS analysis. As shown in Figure 1c, C, Ti, O, and other elements were found in both samples. It can be found that F 1s increases significantly after etching. As can be seen from Figure 1d, a transmission electron microscopy (TEM) image of a single-layer Ti_3_C_2_T_x_ flake obtained from the MXene solution, the longitudinal and lateral dimensions of the sheets are both about a few microns.

**Figure 2:**
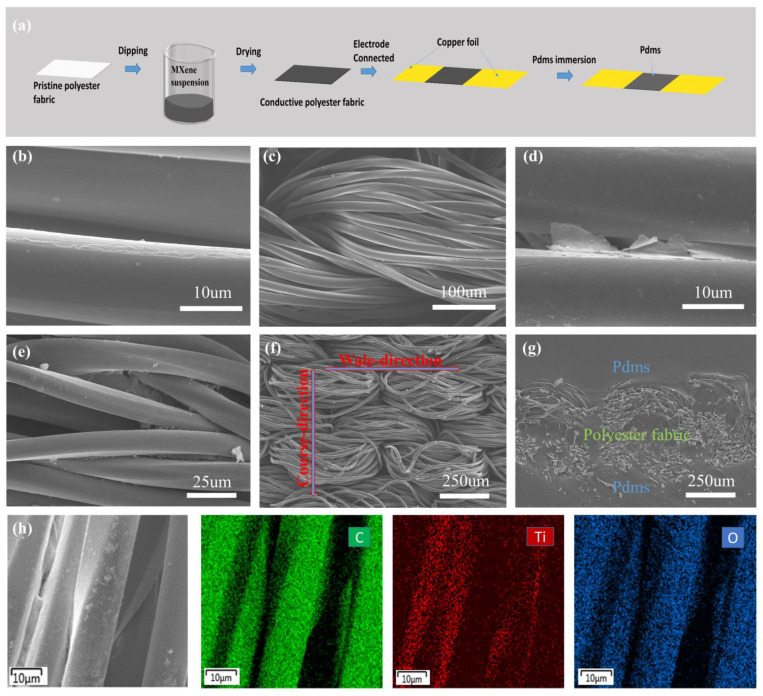
(**a**) Schematic illustration of the PMPF strain sensor fabrication process; (**b**,**c**) SEM images of the surface of the clean polyester fabric at different magnifications; (**d**–**f**) SEM images of the surface of the MXene@polyester fabric at different magnifications; (**g**) SEM image of the fractured surface of the PMPF strain sensor; (**h**) SEM image of the MXene@polyester fabric strain sensor and the corresponding EDX elemental mapping (green: C, red: Ti, blue: O).

Figure 2a illustrates the manufacturing process of the PMPF strain sensor. Figure 2b,c shows SEM images of the cleaned fabric under different magnifications. Figure 2d–f shows SEM images of MXene@polyester fabric at different magnifications. It can be seen that the MXene sheet penetrates the surface and gaps of the weft-knitted polyester fabric to form a conductive network of MXene@polyester fabric. Due to its unique structure, the conductive textile network of MXene can easily withstand course-direction and wale-direction tensile deformation, showing different tensile properties. The SEM of the PMPF cross-section is shown in Figure 2g, which indicates a neatly wrapped PDMS layer around the polyester fiber. The lamination of the PDMS layer protects the internal conductive polyester fibers and maintains the fabric structure after the encapsulation process. MXene@polyester fabric SEM picture and element mapping are shown in Figure 2h. The elements, i.e., C, Ti, and O, can be seen evenly scattered on the polyester fiber’s surface.

**Figure 3:**
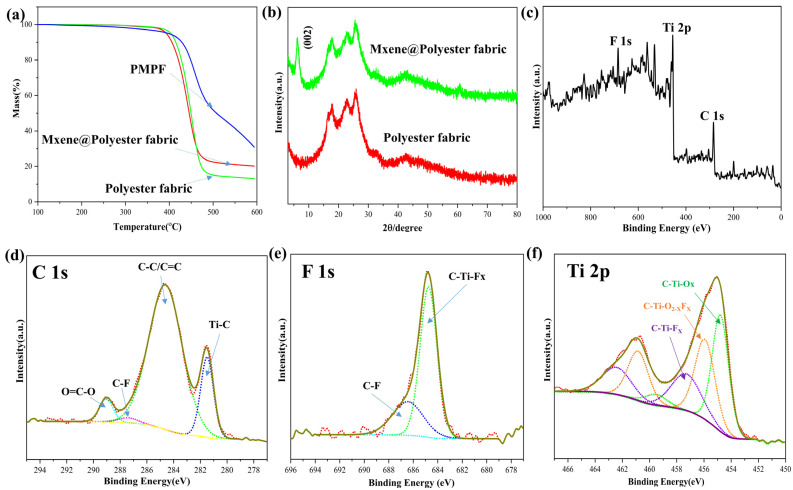
(**a**) TGA data for polyester fabric, MXene@polyester fabric, and PMPF; (**b**) XRD data for polyester fabric and MXene@polyester fabric; (**c**–**f**) XPS data for MXene@polyester fabric.

The TGA curve in Figure 3a clearly illustrates that polyester fabric and MXene@polyester fabric residue tend to be stable at higher temperatures (˃500°). However, PMPF continued to decline after 500 °C. As shown in Figure 3b, the XRD spectra of polyester fabric and MXene@polyester fabric shows that a new diffraction peak appeared at 9.58° (002) in MXene@polyester fabric, indicating that the fabric’s surface has been successfully covered with MXene. Likewise, the chemical structure of Ti_3_C_2_T_x_ on the fabric surface was explored using X-ray photoelectron spectroscopy (XPS). The Ti_3_C_2_T_x_ spectrum presented in Figure 3c confirmed the presence of Ti, C, O, and F elements. The XPS spectra of C1s are depicted in Figure 3d. The peaks at 281.5, 284.6, 287.4, and 289.0 eV correspond to Ti-C, C-C/C=C, C-F, and O=C-O, respectively. Moreover, the XPS spectra of F1s presented in Figure 2e contained two peaks at 684.7 eV (C-Ti-Fx) and 686.4 eV (C-F), and the spectrum of Ti 2p was fitted with three doublets (Ti 2p_3/2_ and Ti 2p_1/2_) as shown in Figure 3f. The peaks at 454.8 and 459.5 eV are attributed to the C-Ti-O_X_ bond, the peaks at 456.1 and 460.9 eV correspond to the C-Ti-O_2−X_F_X_ bond, and the peaks at 457.5 and 462.5 eV correspond to the C-Ti-F_X_ bond. C-Ti-F_X_ has higher binding energy than C-Ti-Ox because F has a higher electronegativity compared to O. This is consistent with the valence state of Ti in the standard Ti_3_C_2_T_x_ [32]. Collectively, these results indicate that large functional groups are present on the surface of Ti_3_C_2_T_x_ (attached to the fabric), which facilitates the bonding between MXene nanosheets and the surface of polyester by electrostatic interaction.

**Figure 4:**
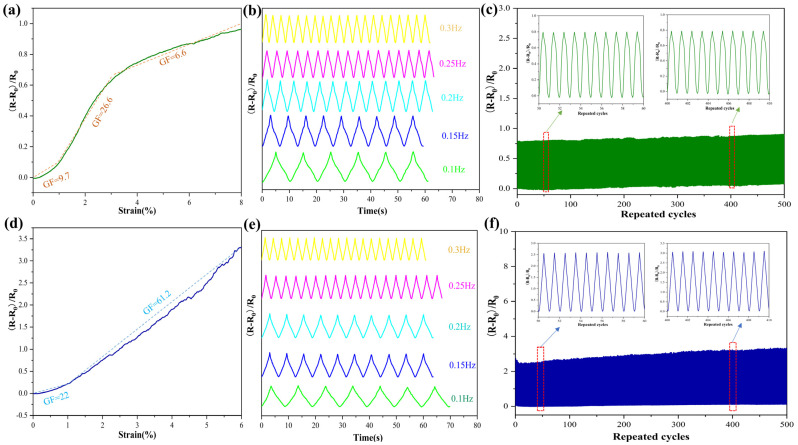
Relative resistance variation of the PMPF sensor to the corresponding applied strain in the (**a**) course direction and (**d**) wale direction. Relative resistance changes at different frequencies of 0.1 to 0.3 Hz due to stretching and releasing in the (**b**) course direction and (**e**) wale direction. Performance of the sensor under 500 cycles of tensile loading in the (**c**) course direction and (**f**) wale direction.

The gauge factor (GF) is widely used to evaluate the sensitivity of a strain sensor and can be calculated by the formula GF = (R − R_0_)/R_0_ε, where R is the resistance under different strains, R_0_ is the resistance without strain, and ε is the strain. Compared with other reported sensors (as shown in Table S1), our sensor prepared by the dipping method shows good tensile properties. Figure 4a shows a typical MXene polyester sensor image with course-direction stretch. As demonstrated in Figure 4a, the polyester sensor showed extensive strain range during the course-direction stretch, and the GF was 9.7 for the strain range within 1%, 26.6 for 1–3% strain range, and 6.6 for 3–8% strain range. Figure 4d shows the performance of the PMPF sensor stretched in the wale direction, showing the GF (22) within a 1% strain range and a comparatively higher GF (61.2) in the range of 1–6% with a good linear response. Thus, Figure 4a,d suggests that the MXene fabric strain sensor provides a wide strain range when stretched in the course direction and high sensitivity when stretched in the wale direction. Moreover, the resistance change in the PMPF strain sensor exhibits almost no frequency dependence when strain is applied at various frequencies (0.1–0.3 Hz) in the course and wale directions, as seen in Figure 4b,e. Furthermore, the corresponding resistance of the PMPF sensors demonstrates excellent stability and good robustness with 5% strain in the course and wale directions after 500 cycles (Figure 3c,f). In conclusion, PMPF strain sensors offer exceptional endurance as well as good sensitivity with a border strain range.

**Figure 5:**
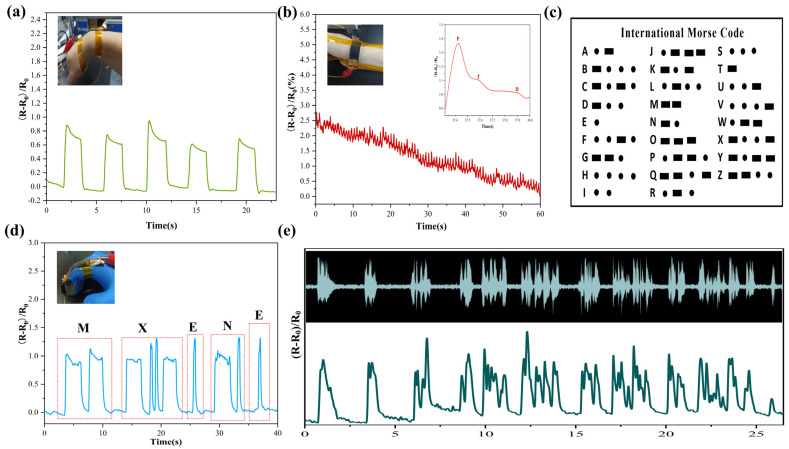
(**a**) Corresponding signals show bending of the wrist. (**b**) Corresponding signals show pulse of the wrist, and inset picture shows one single pulse. (**c**) The International Morse Code. (**d**) The Morse signal (“MXENE”) produced by the bending of the finger. (**e**) Recognition signals compared with the sound from the loudspeaker.

**Figure 6:**
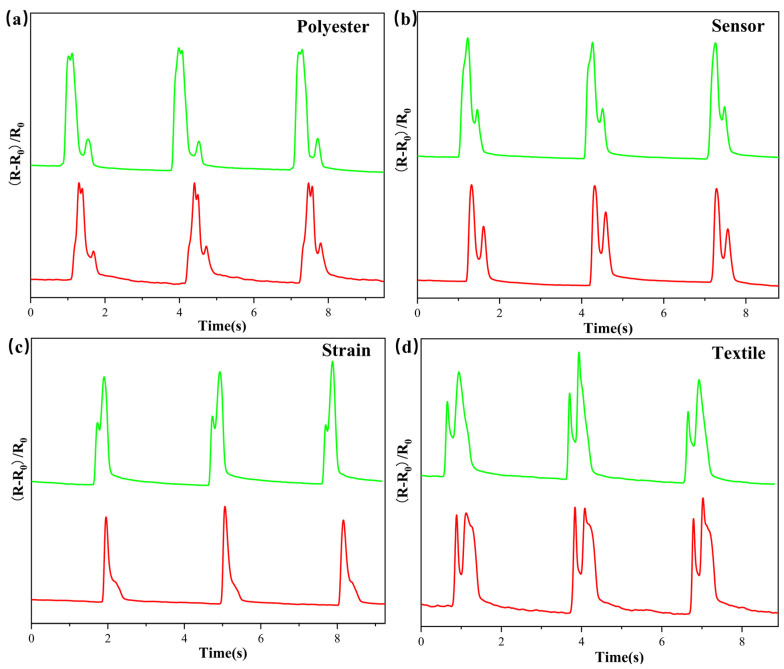
The relative resistance changes of the same word played by the loudspeaker under different pronunciation modes (red color: American English; green color: British English): (**a**) polyester; (**b**) sensor; (**c**) strain; (**d**) textile.

The sensor’s reaction to wrist motion is depicted in Figure 5a, demonstrating that PMPF strain sensors can easily detect human physiological signals. The sensors are tightly attached to the body parts with polyimide (PI) tape. In the process of wrist bending, sensor resistance increases correspondingly. The pulse wave signal contains physiological information such as heart rate, arteriosclerosis, and cardiovascular status, all of which can aid in the prevention of cardiovascular disease. Attaching the sensor to the wrist, as illustrated in Figure 5b, allows the sensor to detect the pulse of the human body. The sensor detects the subject’s pulse at approximately 85 beats/min. The inset illustration shows a single pulse signal with three distinct peaks: “P” (percussion), “T” (tidal), and “D” (diastolic), which confirms the fast response of the sensor and high sensitivity. The PMPF strain sensor can be used for communication based on Morse code, as shown in Figure 5c. A short-term bending of the finger represents a point in the Morse code, and the bending of the finger for a few seconds represents a line in the Morse code. For example, as shown in Figure 5d, the sensor is attached to the finger, and the Morse signal (“MXENE”) is input by bending the finger. The PMPF strain sensor can also monitor the sound signal. Figure 5e (the original audio signal is inset) shows that the sensor based on the PMPF strain sensor is placed directly on the speaker. It was found that the sensor possesses synchronous response characteristics to audio signals and that practically all of the characteristic peaks can be retained. Even when the same word is used, the sensor resistance response induced by different pronunciation styles varies, as illustrated in Figure 6a–d. This result is due to the high sensitivity of the PMPF strain sensor to signals of different sound frequencies and intensities. It can also be further combined with artificial intelligence to accelerate the application development of intelligent sound detectors.

## 4. Conclusions

In summary, MXene was prepared by the MILD method, and the PMPF strain sensor was fabricated by a simple dip-coating method. The MXene was used as a dye for the polyester fabric, giving the MXene textile strain sensor excellent performance. The sensor’s distinct direction sensitivities are due to the variation in course and wale interwoven structure. The maximum GF of the PMPF sensor was 61.2 at a strain of 6% in the wale direction and 26.5 at a strain of 8% in the course direction. In addition, the strain sensor showed outstanding oxidation resistance, stability, and sensitivity. These exceptional characteristics allowed us to detect a wide range of sensing inputs, from minute deformations such as pulse monitoring and sound detection to large-scale muscle movement such as finger bending. Therefore, we are convinced that this multifunctional sensor will be a promising choice for future smart wearable devices, human-machine interaction, and other applications.

## Data Availability

The data presented in this study are available within the article.

## Supplementary Materials

The following supporting information can be downloaded at: https://www.mdpi.com/article/10.3390/nano12050871/s1, Figure S1: The measurement setup for the PMPF strain sensor; Table S1: The comparison of performance parameters between our strain sensor and other reported fabric-based strain sensors [1,16,21,29,33,34,35,36,37,38].
