# Supplementary material for: PDMS-Encapsulated MXene@Polyester Fabric Strain Sensor for Multifunctional Sensing Applications
Source: Nanomaterials (Basel). 2022 Mar 5;12(5):871. doi: 10.3390/nano12050871 (PMC8912599; doi:10.3390/nano12050871)
Supplement: Supplementary file 1 [file nanomaterials-12-00871-s001.zip › nanomaterials-1606403-supplementary.pdf]

Supplementary Materials

PDMS-Encapsulated MXene@Polyester Fabric Strain Sensor for Multifunctional Sensing Applications

Wengang Lu [1,†], Beenish Mustafa [1,†], Zhiyuan Wang [1], Fuzhuo Lian [1] and Geliang Yu [1,2,*]

[1] National Laboratory of Solid State Microstructures and School of Physics, Nanjing University, Nanjing 210093, China; wengang.lu@foxmail.com (W.L.); dg1822501@smail.nju.edu.cn (B.M.); wzy10575@163.com (Z.W.); 15850786328@163.com(F.L.)

[2] Collaborative Innovation Centre of Advanced Microsctructures, Nanjing University, Nanjing 210093, China

* Correspondence: yugeliang@163.com

† These authors contributed equally to this work.

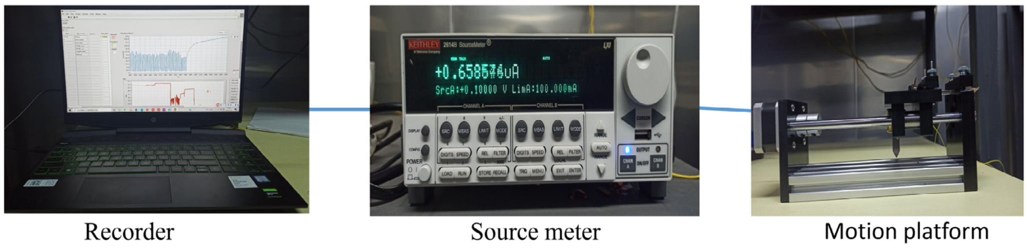

Figure S1: The measurement setup for the PMPF strain sensor

Table S1. The comparison of performance parameters between our strain sensors with other reported fabric-based strain sensors

Sensor composition	Fabrication methods	Strain range	Max Gauge Factor	Refs.
PU/CNT@Fe2+ Fibers	patterning impregnation method	50%	36	[1]
RGO/AgNW/Fabric	Cold spraying method	160%	-0.56	[2]
Graphene-based Fiber	Dipping	100%	10	[3]
Graphene/Polyimide Fabric	a UV laser direct writing technique	4%	27	[4]
Graphene/Polyester	Dipping	15%	-26	[5]
Graphene/Cotton Fabric/Pdms	Dipping	75%	2.49	[6]
Graphene/Silk fabric	Hot press method	10%	124.5	[7]
MXene/Nylon Fabric	Dipping	20%	24.35	[8]
MXene/Polyester/Pva	Layer-by-layer self-assembly	50%	288.43	[9]
MXene/Cotton Fabric	Dipping	15%	4.11	[10]
MXene/Polyester/Pdms	Dipping	8%	61.2	This work

References:

1. Zhang, Z.; Cheng, N.; Zhang, L.; Liu, L. ; Zhao, J.; Yu, H. Wearable strain sensor based on highly conductive carbon nanotubes polyurethane composite fibers. Nanotechnology. 2020, 123803.R1, doi: 10.1088/1361-6528/ab6fdd.

2. Kim, T.; Park, C.; Samuel, E.P.; An, S.; Aldalbahi, A.; Alotaibi, F.; Yarin, A.L.; Yoon, S.S. Supersonically Sprayed Washable, Wearable, Stretchable, Hydrophobic, and Antibacterial rGO/AgNW Fabric for Multifunctional Sensors and Supercapacitors. *ACS Appl. Mater. Interfaces* 2021, *13*, 10013–10025, doi:10.1021/acsami.0c21372.

3. Cheng, Y.; Wang, R.; Sun, J.; Gao, L. A Stretchable and Highly Sensitive Graphene-Based Fiber for Sensing Tensile Strain, Bending, and Torsion. *Adv. Mater.* 2015, *27*, 7365–7371, doi:10.1002/adma.201503558.

4. Park, J.J.; Hyun, W.J.; Mun, S.C.; Park, Y.T.; Park, O.O. Highly stretchable and wearable graphene strain sensors with controllable sensitivity for human motion monitoring. *ACS Appl. Mater. Interfaces* 2015, *7*, 6317–6324, doi:10.1021/acsami.5b00695.

5. Yang, Z.; Pang, Y.; Han, X.L.; Yang, Y.; Yang, Y.; Ling, J.; Jian, M.; Zhang, Y.; Ren, T.L. Graphene Textile Strain Sensor with Negative Resistance Variation for Human Motion

Detection. *ACS Nano* 2018, *12*, 9134–9141, doi:10.1021/acsnano.8b03391.

6. Zheng, Y.; Li, Y.; Zhou, Y.; Dai, K.; Zheng, G.; Zhang, B.; Liu, C.; Shen, C. High-Performance Wearable Strain Sensor Based on Graphene/Cotton Fabric with High Durability and Low Detection Limit. *ACS Appl. Mater. Interfaces* 2020, *12*, 1474–1485, doi:10.1021/acsami.9b17173.

7. Wang, S.; Ning, H.; Hu, N.; Liu, Y.; Liu, F.; Zou, R.; Huang, K.; Wu, X.; Weng, S.; Alamusi Environmentally-Friendly and Multifunctional Graphene-Silk Fabric Strain Sensor for Human-Motion Detection. *Adv. Mater. Interfaces* 2020, *7*, 1–8, doi:10.1002/admi.201901507.

8. Yuan, L.; Zhang, M.; Zhao, T.; Li, T.; Zhang, H.; Chen, L.; Zhang, J. Flexible and breathable strain sensor with high performance based on MXene/nylon fabric network. *Sensors Actuators, A Phys.* 2020, *315*, 112192, doi:10.1016/j.sna.2020.112192.

9. Yu, Q.; Jiang, J.; Su, C.; Huang, Y.; Chen, N.; Shao, H. $Ti_3C_2T_x$ MXene/polyvinyl alcohol decorated polyester warp knitting fabric for flexible wearable strain sensors. *Text. Res. J.* 2021, doi:10.1177/00405175211044163.

10. Liu, L.; Wang, L.; Liu, X.; Yuan, W.; Yuan, M.; Xia, Q.; Hu, Q.; Zhou, A. High-Performance wearable strain sensor based on MXene@Cotton Fabric with network structure. *Nanomaterials* 2021, *11*, doi:10.3390/nano11040889.
